# A novel method for banking stem cells from human exfoliated deciduous teeth: lentiviral TERT immortalization and phenotypical analysis

**DOI:** 10.1186/s13287-016-0309-0

**Published:** 2016-04-04

**Authors:** Zhanhai Yin, Qi Wang, Ye Li, Hong Wei, Jianfeng Shi, Ang Li

**Affiliations:** Department of Orthopedics, First Affiliated Hospital, College of Medicine, Xi’an Jiaotong University, Xi’an, 710061 P. R. China; Department of Periodontology, Stomatological Hospital, College of Medicine, Xi’an Jiaotong University, Xi’an, 710004 P. R. China; Research Center for Stomatology, Stomatological Hospital, College of Medicine, Xi’an Jiaotong University, Xi’an, 710004 P. R. China

**Keywords:** Stem cells from human exfoliated deciduous teeth, Telomerase, TERT, Immortalization, Tumorigenicity

## Abstract

**Background:**

Stem cells from human exfoliated deciduous teeth (SHED) have recently attracted attention as novel multipotential stem cell sources. However, their application is limited due to in vitro replicative senescence. Ectopic expression of telomerase reverse transcriptase (TERT) is a promising strategy for overcoming this replicative senescence. Nevertheless, its potential application and the phenotype as well as tumorigenicity have never been assessed in SHED.

**Methods:**

TERT expression was stably restored in SHED (TERT-SHED) isolated from healthy children aged 6–8 years using lentiviral transduction with a puromycin selection marker. The expression of TERT was detected using reverse transcription polymerase chain reaction, Western blot and immunofluorescence. Surface markers of SHED were detected by flow cytometry. Enzyme-linked immunosorbent assay was used to assess senescence-associated β-galactosidase, while CCK-8 methods were used to examine the proliferation capacity of SHED and TERT-SHED at different passages. Moreover, multilineage differentiation, karyotype, colony formation in soft agar, and tumor formation in nude mice of SHED and TERT-SHED were also examined.

**Results:**

Lentiviral transduction induced stable TERT expression even in SHED at the 40th passage. TERT-SHED showed robust proliferation capacity and low concentration of β-galactosidase. Although they had some different biomarkers than early passage SHED, TERT-SHED at late passage showed similar mutilineage differentiation as TERT at early passage. Moreover, TERT-SHED at late passage showed normal karyotype, no soft agar colony formation, and no tumor formation in nude mice.

**Conclusions:**

TERT-immortalized SHED may be a promising resource for stem-cell therapy, although attention should be paid to the biological behavior of the cells.

## Background

Tissue engineering depends on the association of stem cells, growth factors, organ tissue culture, and tissue engineering materials [1]. In dentistry, regenerative strategies are of great relevance because of hard dental tissue damage, especially as a result of caries lesions, trauma, or iatrogenic procedures. The principles of regenerative medicine can be applied to endodontic tissue engineering. Regeneration of the pulp-dentin complex will allow natural replacement of damaged or missing tooth structures through the activation of tissue-specific stem cells of animal origin or transplantation of stem cells isolated and ex vivo expanded [2]. Stem cells can be found to be quiescent in their niche in all tissues of an adult organism. In response to organ injury, these cells initiate their proliferation and differentiation with the aim of healing injured tissue. Among various dental pulp stem cells (DPSCs), stem cells from human exfoliated deciduous teeth (SHED) have recently attracted attention as novel multipotential stem cell sources [3]. The loss of primary teeth creates the perfect opportunity to recover and store this convenient source of stem cells. Isolating SHED is simple, painless and convenient, and involves little or no trauma. These immature stem cells are important in the regeneration and repair of craniofacial defects, tooth loss, and bones because of their capability to proliferate and differentiate [4]. SHED generate rapidly and grow faster than adult stem cells, thus suggesting that they are less mature. SHED are postnatal stem cells capable of differentiating into osteogenic, odontogenic, adipogenic, and neural cells [5].

Although they have high self-renewal capacity, dental stem cells such as SHED will ultimately enter an irreversible proliferation-arrested state referred to as replicative senescence after long term in vitro culture under culture stresses, such as hyperoxia and elevated temperature [6]. Several strategies have been explored to overcome this replicative senescence, such as treatment of SHED with benzopyrene (BaP), radiation, or ectopic expression of viral oncogenes [7]. Long-term treatment with carcinogenic agents such as BaP results in transformation of cells as evidenced by chromosomal abnormalities, anchorage-independent growth in soft agar, and tumorigenicity in nude mice [8]. Radiation has been shown to be sufficient for immortalization of breast epithelial cells [9]. However, immortalization by radiation occurs relatively infrequently and results in morphological transformation of cells [9] and formation of tumors in nude mice [10]. A number of viral oncogenes, including simian virus-40 (SV40) large T-antigen, adenovirus E1A and E1B, polyoma T-antigen, and papillomavirus E6 and E7, have also been used to immortalize human cells [11–13]. Although immortalized cell lines have been successfully established by transfecting cells with viral oncogenes, inactivation of protein products of the tumor suppressor p53 and retinoblastoma (Rb) [14, 15], introduction of karyotypic instability, and transformation of phenotype have been reported in many studies [16, 17].

The cellular senescence and the lifespan depend on the loss rate of telomeres during each cell division and the primary length of the telomere [18]. Telomerase reverse transcriptase (TERT; catalysis subunit of telomerase) plays critical roles in the maintenance of telomere length during cell division [19–21]. It has been demonstrated that telomerase reconstitution via TERT expression could extend the telomere, prolong the lifespan of cells, and even immortalize cells [22, 23]. It has been established that the expression of TERT is a key step in human cellular proliferation, differentiation, and apoptosis. Moreover, recent findings indicate that TERT regulates stem cell properties in stemness sustaining and self-renew characterizations [24, 25]. While ectopic expression of TERT does significantly lengthen the lifespan of cells, enhanced telomerase activity is also a feature of many types of tumors and malignancies [26, 27]. The potential tumorigenicity of TERT-expressed stem cells remains controversial [27–29]. Therefore, the tumorigenicity of TERT expression in human stem cells needs to be further validated.

Our primary goal in this study was to create an immortalized SHED cell line by stable expression of TERT. Moreover, we assessed the multipotency and the potential tumorigenicity of our immortalized SHED cell line.

## Methods

### Subjects and cell culture

The SHED were obtained from the deciduous teeth of children aged 6–8 years. Every patient involved in the study consented to participate in the study and signed the paper consent. This study was approved according to guidelines set by the Ethic Committee of the Dental Hospital, Xi’an Jiaotong University. The deciduous anterior teeth used in this study were near natural exfoliation, with less than one third of the root remaining, and without any deep caries, restoration, periapical lesions, or internal resorption. After extraction, pulp tissues from the deciduous teeth were extirpated using a barbed broach (Mani, Utsunomiya Toshi-ken, Japan), washed with phosphate-buffered saline (PBS; Invitrogen, Carlsbad, CA, USA), and then treated with collagenase type I (3 mg/ml; Invitrogen) and dispase (4 mg/ml; Invitrogen) for 30 min at 37 °C; they were then filtered through a 70-μm cell strainer. The SHED were cultured in a DMEM/F12 medium supplemented with 15 % fetal bovine serum, 2 mmol/l L-glutamine, 100 μmol/l L-ascorbic acid-2-phosphate, 100 U/ml penicillin, 100 μg/ml streptomycin, and 0.25 μg/ml amphotericin B. After 7 days, cell colonies were observed. Individual cell colonies were collected by the filter paper enzyme digestion method. The derived cells were SHED.

### Cloning of TERT in lentiviral expression plasmid and lentiviral production

pCMV6-XL5 plasmid (OriGene Technologies, Beijing, China) containing full-length cDNA of human TERT (3.6 kb) [Genbank:NM_198253.2] was amplified in DH5α *E. coli* strain. The cDNA clone of TERT and GV166 lentiviral vector (GeneChem Co., Ltd., Shanghai, China) were digested by a cocktail of *EcoR* I and Sal I (New England Biolabs, Ipswich, USA). The subsequent fragments were purified and recombined by T4 ligase (New England Biolabs) and then transformed into DH5α *E. coli* selecting for ampicillin resistance. The transformants were screened for correct insertion/orientation of the TERT fragment by restriction analysis. GV166 vector not recombined with TERT was used as the control vector. For lentiviral production, the GV166-TERT or control plasmid was co-transfected into 293FT cells with Lenti-Easy Packaging Mix (GeneChem Co., Ltd.) at a 1:3 ratio using Lipofectamine™ reagent (Invitrogen). Forty-eight hours after transfection, the virus-containing supernatant was harvested and stored in aliquots at −80 °C. All cell culture procedures were performed under biosafety level 2 conditions.

### Transduction of SHED with lentiviral vectors

Cells were plated 24 h before transduction at a density of 5 × 10^4^ cells per well in six-well plates in the presence of 5 μg/ml polybrene. Transduction of SHED was carried out with TERT or control lentivirus at a multiplicity of infection (MOI) of 65. Transduced cells were passaged, and selected with puromycin (1.5 mg/ml) for 5 days.

### Extraction of total RNA and RT-PCR

Total RNA was extracted using TRIzol reagent (Invitrogen, Carlsbad, CA, USA) according to the manufacturer’s protocol, and RNase-free DNase I was used to remove DNA contamination. Reverse transcription (RT) was performed with 2 μg total RNA using M-MLV Reverse transcriptase (Promega, Madison, WI, USA) to synthesize first-strand cDNA according to the manufacturer’s recommendation, followed by cDNA amplification using the specific primers for *TERT* and the β-actin primer. Primers used in this study were as follows: 5′-AGAGTGTCTGGAGCAAGTTG-3′ (forward) and 5′-GGATGAAGCGGAGTCTGG-3′ (reverse) for *TERT*; 5′-ATCGTGCGTGACATTAAGGAGAAG-3′ (forward) and 5′-GAGGAAGGAAGGCTGGAAGAGTG-3′ (reverse) for β-actin; and the corresponding polymerase chain reaction (PCR) products were 140 bp and 179 bp, respectively.

### Western blot

Cells were lysed in RIPA lysis buffer, and the lysates were harvested by centrifugation (13,523 × g) at 4 °C for 30 min. Approximately 20 μg protein samples were then separated by electrophoresis in a 12 % sodium dodecyl sulfate polyacrylamide gel and transferred onto a polyvinylidene fluoride membrane. After blocking the non-specific binding sites for 60 min with 5 % non-fat milk, the membranes were incubated overnight at 4 °C with a mouse monoclonal antibody against human TERT (Abgent, USA, at a 1:1000 dilution). The membranes were then washed three times with TBST (tris-buffered saline with tween-20) for 10 min and probed with the horseradish peroxidase (HRP)-conjugated rabbit anti-mouse IgG antibody (Immunology Consultants Laboratory, USA, at a 1:2000 dilution) at 37 °C for 1 h. After three washes, the membranes were developed by an enhanced chemiluminescence system (Cell Signaling Technology, Danvers, MA, USA). A rabbit polyclonal antibody against human β-actin (Abcam, UK, at a 1:10000) was set as the inner control.

### Immunofluorescence staining

Cells were fixed in 4 % paraformaldehyde, permeabilized with 0.1 % Triton1-X100, and blocked with 10 % horse serum in PBS for 1 h. The TERT antibody was prepared at 1:100 dilution and further incubated with the samples for 18 h at 4 °C. After washing with PBS, the cell was incubated with FITC-labeled goat antimouse antibody (Invitrogen) at 1:500 dilution for 45 min. Cells were also counterstained with DAPI. The fluorescence was evaluated by fluorescence microscope (Apotome).

### Detection of surface markers by flow cytometry

The stem cell nature of SHED was analyzed using flow cytometry. Cells cultured with basal medium before cell differentiation were harvested using trypsin and washed twice with PBS. For cell surface staining, cells were fixed, washed and incubated with FITC-conjugated monoclonal or polyclonal antibodies against CD34, CD45, CD146 or STRO-1 (all Biolegend, CA, USA). For intracellular staining, cells were fixed and permeabilized using the Fix & Perm kit (Invitrogen), then washed and incubated with FITC-conjugated monoclonal or polyclonal antibodies against Oct-4 or Nanog (both Biolegend). FITC-conjugated IgG (Biolegend) was used as a negative control. The cells were analyzed using a FACScan flow cytometer (Becton Dickinson, Franklin Lakes, NJ, USA).

### Multilineage differentiation assays

In vitro osteogenic differentiation of SHED was performed as previously published [30] using 100 nM dexamethasone, 10 mM β-glycerophosphate, and 50 M L-ascorbic acid-2-phosphate (Sigma). A total of 5 × 10^3^ cells/well were seeded on to a six-well plate. After 16 days cells were assayed by von Kossa staining using a standard protocol. Adipogenic differentiation was accomplished as previously published [30] by 1 M dexamethasone, 0.2 mM indomethacin, 0.1 mg/ml insulin, and 1 mM 3-isobutyl-1-methylxanthin (IBMX) (Sigma). The maintenance medium consisted of 0.1 mg/ml insulin in standard medium. A total of 4 × 10^3^ cells/well were seeded on to a 12-well plate. Stimulation was started when cells reached full confluency. Cells were grown for 5 days in induction medium and thereafter for 2 days in maintenance medium, and were then switched to induction medium again. After 16 days of stimulation, the cells were assayed by oil red staining using a standard protocol. Chondrogenic differentiation was achieved in aggregate cultures as previously published [30] with 100 nM dexamethasone, 1 mM pyruvate, 195 M L-ascorbic acid-2-phosphate, 350 M L-proline, 1.25 % (v/v) insulin-transferrin-selenious acid mix (ITS, 100×), 5.35 g/ml linolic acid, 1.25 mg/ml bovine serum albumin (BSA; Sigma), and transforming growth factor-3 (TGF-3, 10 ng/ml; R&D Systems, Minneapolis, MN, USA). A total of 2.5 × 10^5^ cells were used per pellet. Sections (12 μm) were cut with a cryostat vacutome HM 200 OM (Microm, Walldorf, Germany). Anionic sulfated proteoglycans were detected by toluidine blue metachromasia. Slices were stained in 1 % toluidine blue solution (Sigma, Munich, Germany) and 1 % sodium tetraborate (Sigma).

### Real-time PCR

Total RNA and cDNA of differentiation-induced cells were prepared according to the above-mentioned protocols. Differentiation markers and GAPDH were amplified by quantitative real-time PCR using the following primers: *ALP* forward 5′-CATGCTGAGTGACACAGACAAGAA-3′, reverse: 5′-ACAGCAGACTGCGCCTGGTA-3′; *BSP* forward 5′-CTGGCACAGGGTATACAGGGTTAG-3′, reverse: 5′-GCCTCTGTGCTGTTGGTACTGGT-3′; *LPL* forward: 5′-GTCACGGGCTCAGGAGCATTA-3′, reverse: 5′-GCTCCAAGGCTGTATCCCAAGA-3′; *PPAγ-2* forward: 5′-GCTCTGCAGGAGATCACAGA-3′, reverse: 5′-GGGCTCCATAAAGTCACCAA-3′; *ACAN* forward: 5′-ACGAAGACGGCTTCCACCAG-3′, reverse: 5′-TCGGATGCCATACGTCCTCA-3′; *COL2A1* forward: 5′-CCAGTTGGGAGTAATGCAAGGA-3′, reverse: 5′-ACACCAGGTTCACCAGGTTCA-3′; *GAPDH* forward: 5′-CTCCTCCTGTTCGACAGTCAGC-3′, reverse: 5′-CCCAATACGACCAAATCCGTT-3′. Gene-specific amplification was performed in an ABI 7900HT real-time PCR system (Life Technologies, Carlsbad, CA) with a 15-μl PCR mix containing 0.5 μl cDNA, 7.5 μl 2 × SYBR Green master mix (Invitrogen), and 200 nM of the appropriate primers. The mix was preheated at 95 °C for 10 min and then amplified in 45 cycles of 95 °C for 30 s and 60 °C for 1 min. The resolution curve was measured at 95 °C for 15 s, 60 °C for 15 s, and 95 °C for 15 s. The Ct (threshold cycle) value of each sample was calculated, and the relative mRNA expression was normalized to the GAPDH value (2^–ΔΔCt^ method). The final expression value of differentiation markers was standardized according to that of control cultures.

### Senescence-associated β-galactosidase assay by ELISA

Cells (1 × 10^6^) were lysed and the supernatant was collected by centrifuge. The activity of β-galactosidase (β-GAL) in SHED was assessed using the human β-GAL enzyme-linked immunosorbent assay (ELISA) Kit (CSB-E09463h, Cusabio, China) according to the manufacturer’s recommendations.

### Proliferation assay

Cells were plated at a density of 1 × 10^3^/well in 96-well plates and cultured in basal medium. A CCK-8 assay was performed twice a day according to the cell counting kit protocol (Keygen Biotech, Nanjing, China) for 12 consecutive days. The values for each well were spectrophotometrically measured at 450 nm.

### Cytogenetic analysis

Metaphase spreads were prepared from exponentially growing TERT-SHED at various passages. Cells were harvested and fixed following standard protocols [31]. Chromosome analysis was performed using the GTG-banding technique [31]. Fifteen metaphases captured by a CCD camera were analyzed and karyotyped using the CytoVision system (Leica Biosystems, Nussloch, Germany). Chromosome identification and karyotype description were made in accordance with the International System for Chromosome Nomenclature [32].

### DNA isolation and PCR analysis of *CDKN2A*

Genomic DNA from SHED P4, and TERT-SHED P20 and P40 was extracted using the QIAamp DNA Mini Kit (QIAGEN, Duesseldorf, Germany) according to the manufacturer’s recommendations. The exon 2 of the *CDKN2A* gene was amplified in an ABI GeneAmp 9600 PCR system using the following primers: forward: 5′-CCTGGCTCTGACCATTCTGTTC-3′, reverse: 5′-GCTTTGGAAGCTCTCAGGGTAC-3′. The PCR mixture contained 100 ng genomic DNA, 10 μl 2 × Taq PCR Master Mix (TIANGEN Biotech, Beijing, China), and 0.1 pmol/μl of each primer in a total 20 μl volume. The PCR cycling conditions were 94 °C (2 min) for 1 cycle, 94 °C (30 s), 55 °C (40 s), 72 °C (2 min) for 36 cycles, and a final extension of 72 °C (10 min). The PCR products were 386 bp in length.

### Soft agar assay

TERT-SHED or tongue cancer cells (Tca-8113; 2500 cells) in logarithmic growth phase were trypsinized and suspended into a single cell suspension in 0.5 ml 0.8 % top agar solution (37 °C). The cells were aliquoted on the top of a pre-prepared 1 ml 1.2 % base agar layer, and then incubated for 28 days at 37 °C with 5 % CO_2_. The well plates were stained with 0.005 % crystal violet for 2 h before a photograph was taken under a microscope [33].

### Tumorigenicity in nude mice

Twenty athymic nude mice were divided into four groups: SHED P2, TERT-SHED P20, Tca-8113, and PBS control. Cells (2 × 10^6^) of each cell type were suspended in 200 μl PBS and injected subcutaneously into the fore and hind limb armpit under general anesthesia. Mice were sacrificed after 8 weeks by CO_2_ overdose. All procedures were performed according to animal protection legislation and approved by the Ethics Committee of the Dental Hospital, Xi’an Jiaotong University. Photographs were taken every week for macroscopic evaluation. Skin and underlying soft tissue of the relevant area were dissected 8 weeks after cell implantation. Slides were prepared and stained with hematoxylin and eosin dye, and investigated for possible tumor growth [34].

### Statistical analysis

The data for β-GAL concentration and proliferation were expressed as mean ± standard deviation (SD). One-way proliferation data were used to compare the difference in β-GAL and followed by Fisher’s LSD post hoc test. Repeated measurement analysis of variance was used to compare the differences between proliferation curves. All statistical analyses were performed using IBM SPSS Statistics 19.0 software and a *P* value <0.05 was considered significant.

## Results

### SHED morphology

**Fig. 1:**
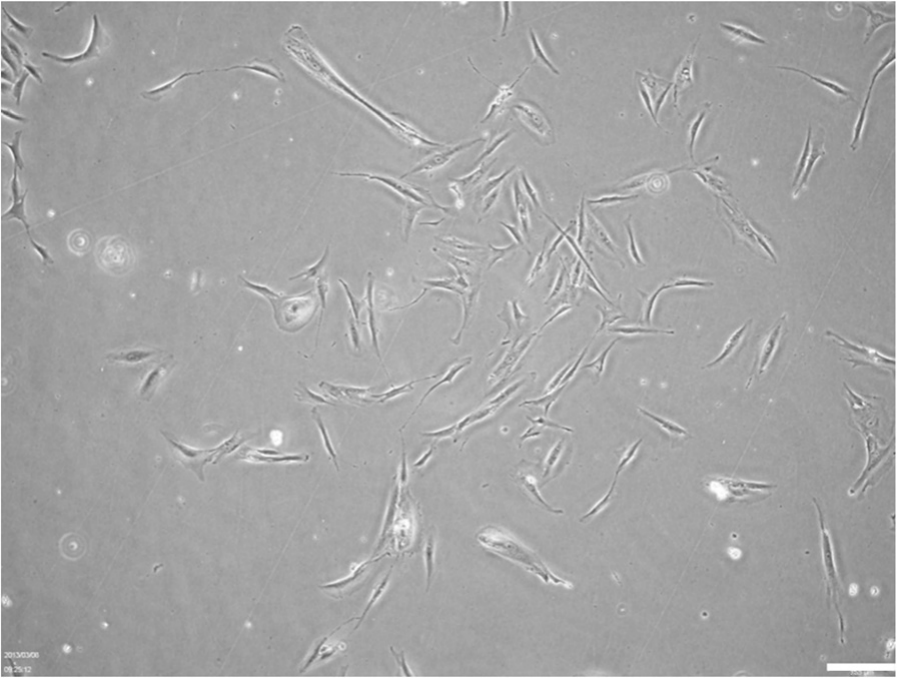
Typical cell morphology of SHED under phase contrast microscope. SHED revealed a typical fibroblast-like morphology. *Scale bar* = 50 μm

The morphology of SHED was analyzed under phase contrast microscope (Fig. 1). SHED spread along the surface of the culture plates, showing rapid growth within the first week. Cell colonies were seen from the fifth day, as the cells grew to confluence at 10–14 days.

### Expression of TERT in SHED after lentiviral transduction

**Fig. 2:**
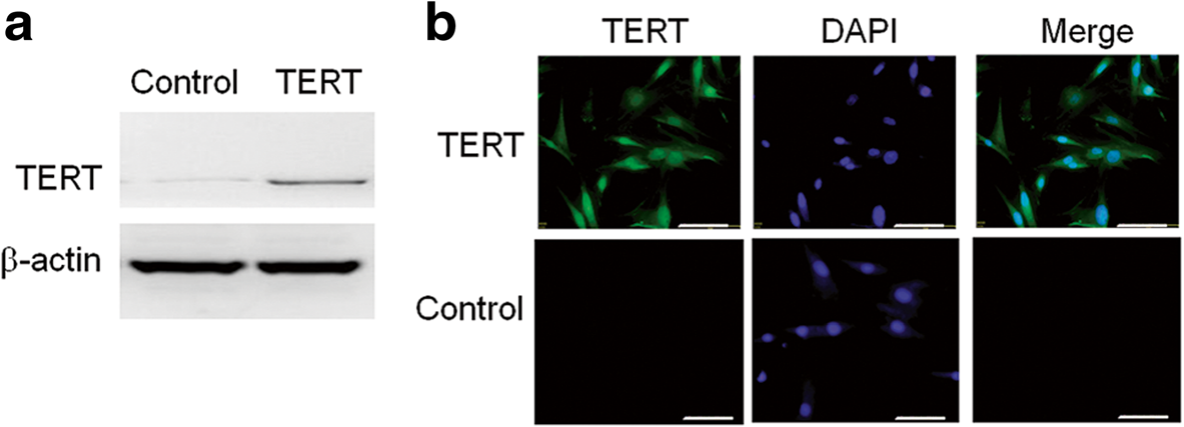
Expression of TERT assessed by Western blot (**a**) and immunofluorescence (**b**) in SHED at the 20th passage 60 h after control and lentiviral transduction. Transduction of TERT-recombined lentivirus restored the expression of TERT in SHED and the optimal MOI was 65. *Scale bar* = 50 μm. *TERT* telomerase reverse transcriptase

We first analyzed the expression of TERT in SHED after stable lentiviral transduction using Western blot and immunofluorescence. As shown in Fig. 2, TERT transduction remarkably restored the expression of TERT in SHED (TERT-SHED), while the control virus did not show TERT expression (SHED).

### Expression of TERT in TERT-SHED during passage

**Fig. 3:**
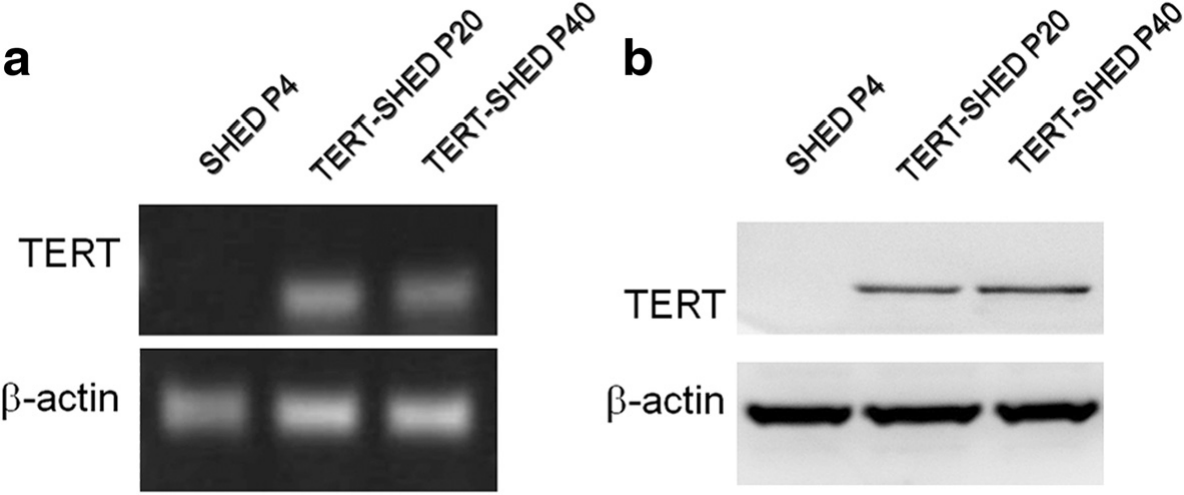
Expression of TERT assessed by RT-PCR (**a**) and Western blot (**b**) in SHED and TERT-SHED at different passages. SHED showed robust mRNA and protein expression of TERT even at the 40th passage after lentiviral transduction. *P* passage, *SHED* stem cells from human exfoliated deciduous teeth, *TERT* telomerase reverse transcriptase

We further analyzed the expression of TERT during the passage of TERT-SHED using RT-PCR and Western blot. As shown in Fig. 3, TERT expression remained stable even in the 40th passage.

### Surface markers of SHED

**Fig. 4:**
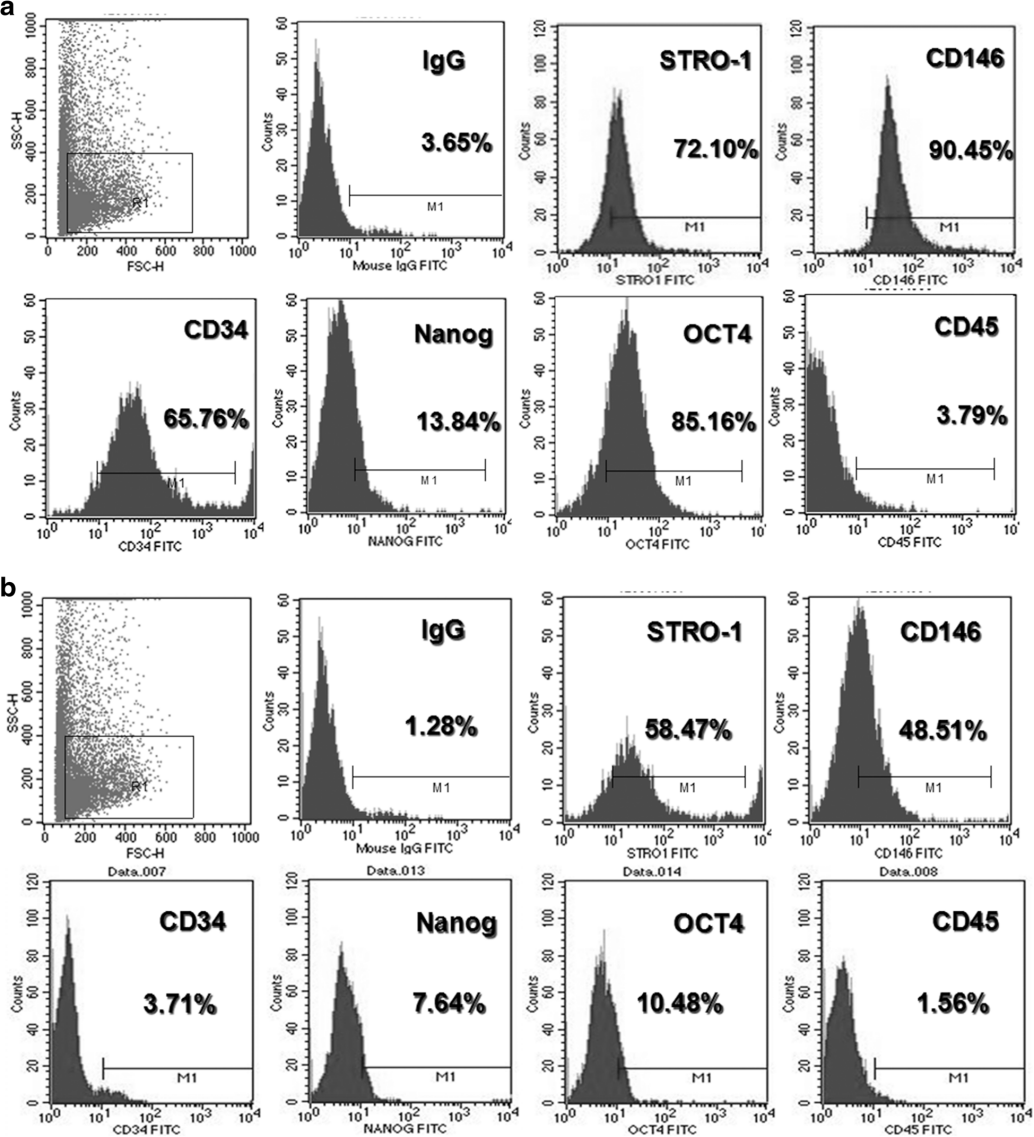
Surface marker of SHED P4 (**a**) and TERT-SHED P20 (**b**) as assessed by flow cytometry. TERT-SHED retained the expression of stem cell markers, such as STRO-1, CD146, and Nanog, at the 20th passage. However, the expression of CD34 and OCT4 was downregulated at the 20th passage, whereas the expression of CD45 remained at a low level

The flow cytometry analysis was applied to quantify the expression ratios of specific surface antigens in SHED and TERT-SHED. The SHED at the fourth passage (SHED P4) showed robust expression of CD146 (90.45 %), STRO-1 (72.10 %), CD34 (65.76 %), and Oct-4 (85.16 %), weak expression of Nanog (13.84 %), and nearly negative expression of CD45 (3.79 %) (Fig. 4a). The TERT-SHED at the 20th passage (SHED P20) showed decreased expression of CD146 (48.51 %), STRO-1 (58.47 %), Oct-4 (10.48 %), Nanog (7.64 %), and CD34 (3.71 %) (Fig. 4b).

### Multilineage differentiation of SHED

**Fig. 5:**
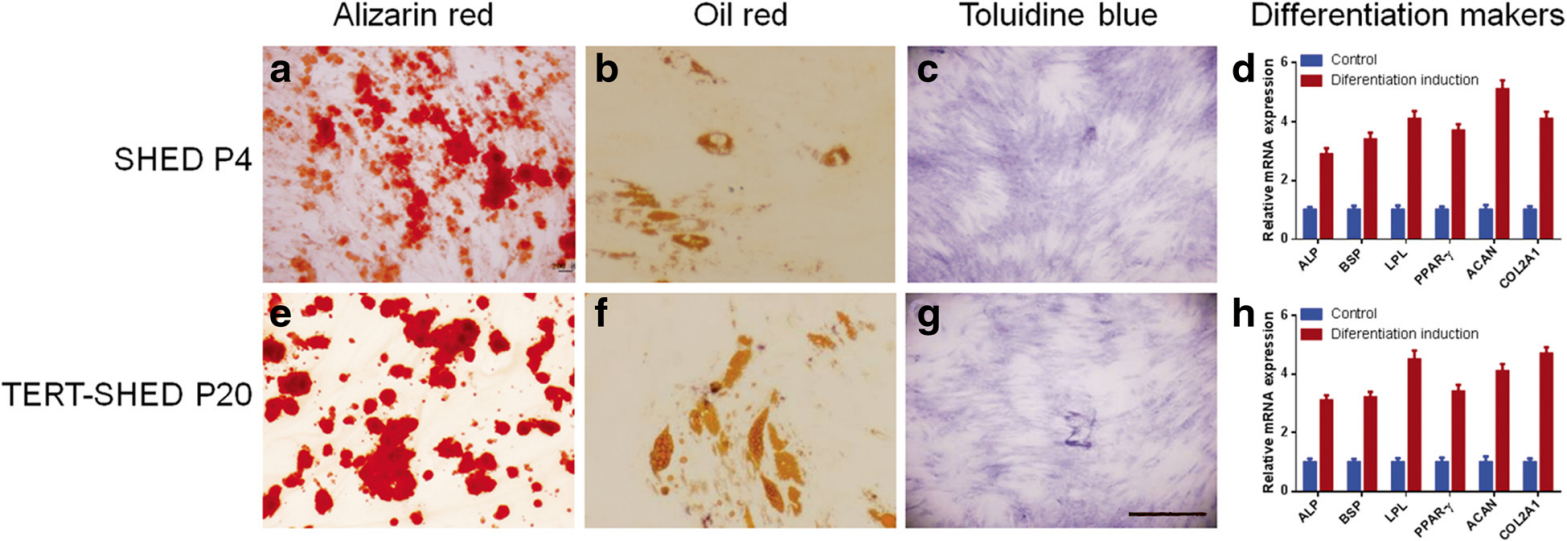
Multilineage differentiation assay of SHED P4 and TERT-SHED P20. Calcium deposition around cells was stained red by Alizarin Red S (**a** and **e**) after induction; adipose droplets in cells were stained orange by Oil Red O after adipogenic induction (**b** and **f**); proteoglycans in cells were stained blue by Toluidine blue after chondrogenic induction (**c** and **g**). The expression of osteogenic, adipogenic, and chondrogenic differentiation markers were examined using real-time PCR and standardized according to that of control culture (**d** and **h**). TERT-SHED P20 showed similar osteogenic, adipogenic, and chondrogenic differentiation to SHED P4. *Scale bar* = 50 μm. *P* passage, *SHED* stem cells from human exfoliated deciduous teeth, *TERT* telomerase reverse transcriptase

The multilineage differentiation potential of SHED was measured by differentiation induction assay. SHED P4 could different into osteogenic, adipogenic, and chondral cell lineages, as revealed by positive staining for Alizarin Red S, Oil Red O and Toluidine blue, respectively (Fig. 5a–c). Moreover, TERT-SHED P20 showed similar differentiation capacity to SHED P4 (Fig. 5e–g). Furthermore, real-time PCR analysis confirmed that TERT-SHED P20 and SHED P4 had similar expression of osteogenic (ALP and BSP), adipogenic (LPL and PPAR-γ), and chondrogenic (ACAN and COL2A1) differentiation markers (Fig. 5d and h).

### Cell senescence and proliferation capacity of SHED

**Fig. 6:**
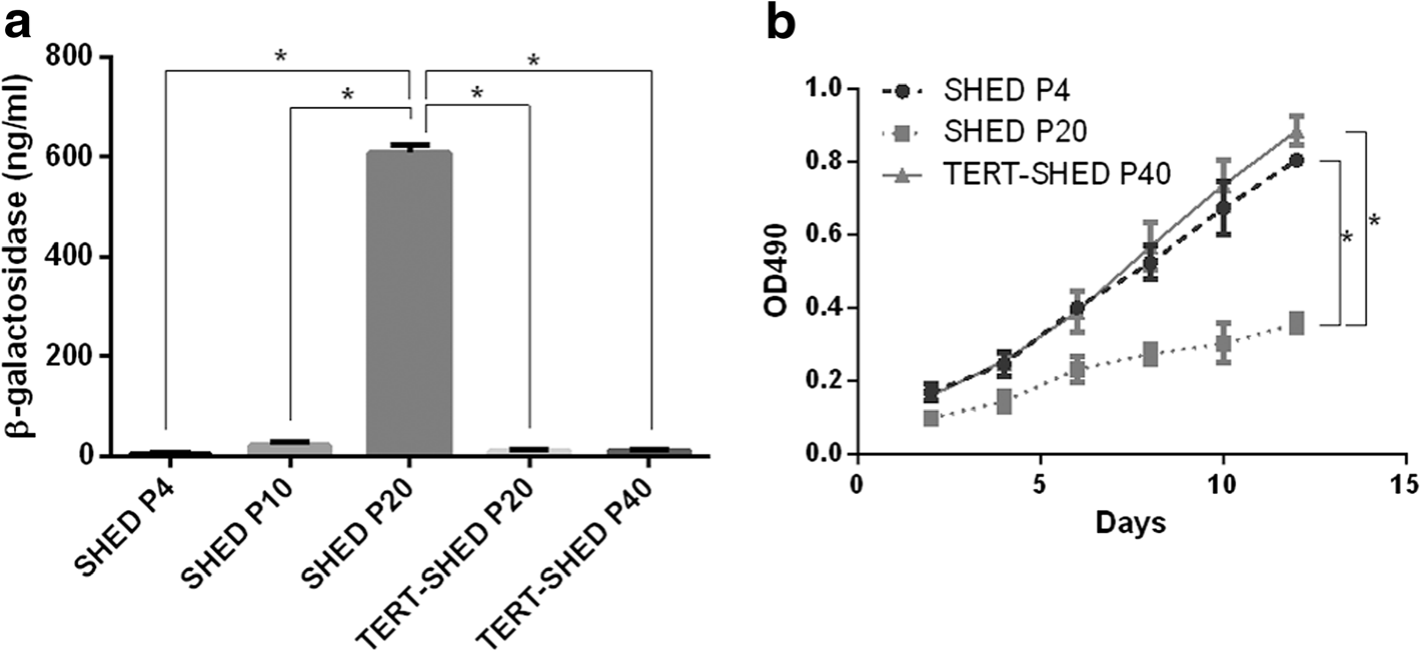
Comparison of β-GAL expression and proliferation capacity of SHED and TERT-SHED at different passages. **a** TERT-SHED showed low β-GAL expression at a late passage (40th passage; *P40*), whereas SHED P20 showed senescence as indicated by remarkably high β-GAL expression. **b** TERT-SHED at late passages (20th and 40th passages; *P20* and *P40*) showed a significantly stronger capacity for proliferation than SHED at the fourth passage (*P4*). *P < 0.05. *OD* optical density, *SHED* stem cells from human exfoliated deciduous teeth, *TERT* telomerase reverse transcriptase

β-GAL activity at pH 6 is a known characteristic of senescent cells which is not found in presenescent, quiescent or immortal cells. We first examined the senescence marker, β-GAL, in SHED and TERT-SHED at different passages. As shown in Fig. 6a, the concentration of β-GAL in the 20th passage of SHED (SHED P20) was as 120 times that in SHED P4. However, β-GAL concentration remained at a very low level in the 40th passage of TERT-SHED (TERT-SHED P40). The proliferation capacity of SHED was detected by CCK-8 assay. As shown in Fig. 6b, the proliferation capacity of SHED at late passage (P20) significantly decreased. However, TERT-SHED P40 had similar proliferation potential to the early passage (P4) of SHED.

### Cytogenetic of TERT-SHED

**Fig. 7:**
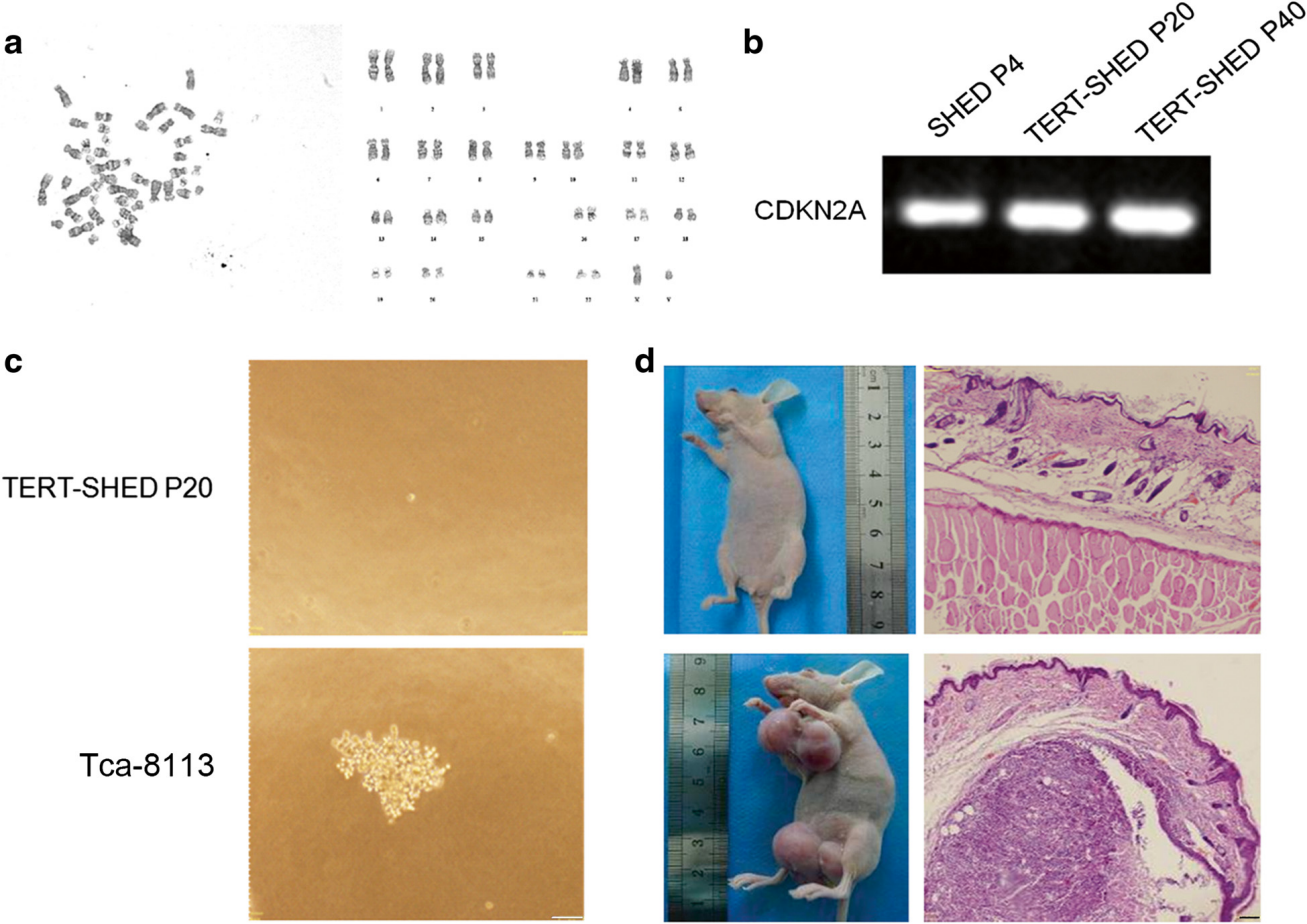
Assessment of potential tumorigenicity of TERT-SHED. **a** TERT-SHED P40 showed no abnormality of karyotype. **b** PCR application showed that there was no deletion of CDKN2A in SHED P4, TERT-SHED P20 or TERT-SHED P40. **c** TERT-SHED P20 showed no colony formation in soft agar, with Tca-8113 cancer cells as a positive control. *Scale bar* = 50 μm. **d** TERT-SHED P20 showed no tumorigenicity in nude mice, with Tca-8113 cells as a positive control. *Scale bar* = 50 μm. *P* passage, *SHED* stem cells from human exfoliated deciduous teeth, *TERT* telomerase reverse transcriptase

Atypia is one of the major characteristics of cancer. We therefore analyzed the karyotype of TERT-SHED at late passage (P20). As shown in Fig. 7a, no polyploid mutation or chromosomal deletion was found in TERT-SHED P40.

### Integrity of genomic *CDKN2A*

Since a deletion of the *CDKN2A* gene locus has been described after ectopic TERT expression using retroviral vectors [35], we analyzed the integrity of *CDKN2A*. PCR amplification yielded a band of the expected size for SHED P4, TERT-SHED P20 and TERT-SHED P40 (Fig. 7b).

### Colony formation of TERT-SHED

The soft agar colony formation assay is a common method to monitor anchorage-independent growth. We thus examined the colony formation of TERT-SHED P20. As shown in Fig. 7c, only a single cell was noticed growing in soft agar in the culture of TERT-SHED P20, while cell aggregates were formed in the culture of tongue cancer cells (Tca-8113).

### Tumorigenicity of TERT-SHED in nude mice

We further assessed tumor formation of TERT-SHED P20 in nude mice. As shown in Fig. 7d, no tumor formation was seen in TERT-SHED P20. However, tumor formation was noticed after Tca-8113 cell inoculation.

## Discussion

In this study, we established a method to immortalize SHED using ectopic stable expression of TERT by lentiviral vector. We found that TERT-SHED showed a robust proliferation capacity even in late passages without cell senescence as indicated by low activity of β-GAL. Although they had some different biomarkers compared to early-passage SHED, TERT-SHED at late passage showed similar mutilineage differentiation to TERT at early passage. We also assessed the potential tumorigenicity of TERT-SHED, and found that TERT-SHED at late passage showed low tumorigenicity, as indicated by normal karyotype, no soft agar colony formation, and no tumor formation in nude mice. These data suggest that TERT expression may be a safe technique for banking SHED for tissue repair.

SHED are mesenchymal-like cells and are an attractive candidate for use in tissue repair thanks to their multipotentiality, easy availability, and immunoprivileged status [36]. They do not induce an allogenic reaction and may even suppress host T-cell proliferation [37], suggesting that cells cultured from a single donor may be expanded in vitro to form a reserve pool that could be used for multiple recipients. However, during in vitro culturing, SHED undergoes replicative senescence and loses its ability to differentiate over time [38]. Thus, immortalization of dental stem cells (DSCs) and establishment of a dental stem cell line are important for DSC research and regenerative dentistry.

It is generally thought that replicative senescence of stem cells is a result of genetic instability after critical shortening of telomeres [39]. Telomerase had the enzymatic activity to maintain and elongate telomere length during cell division. Ectopic expression of TERT has been proven in many studies to maintain the telomere length in different types of cells, thus immortalize cells and prevent cells from loss of function. Using this approach, stable expression of TERT prevents replicative senescence in human mesenchymal stem cells (hMSCs) [40–43], with a lifespan extension of more than 3 years [44]. These findings are consistent with our results that stable TERT expression causes a continuous proliferation of SHED with a lack of senescence-associated β-GAL staining in robust cells even in the 40th passage, whereas untransduced cells went into senescence in the 20th passage, indicating that TERT expression may be a useful strategy for immortalizing stem cells.

Usually, long-term in vitro culture of stem cells results in impaired differentiation capacity [45]. Previous studies have demonstrated that MSCs overexpressing TERT exhibit an increased osteogenic differentiation potential [45], while telomerase deficiency impairs differentiation of hMSCs [46]. In our study, although TERT-SHED at late passage showed some decrease in the biomarkers, its differentiation into osteal, adipic, and chondric cells was similar to that in SHED at early passage, suggesting that telomere length maintenance plays an important role in the differentiation of stem cells.

With the success of immortalization of stem cells by TERT, concerns about potential malignant transformation by viral TERT transduction were raised. It has been reported that long-term culture of TERT-transduced adult MSCs using a retrovirus resulted in neoplastic transformation [26, 35]. Insertional mutagenesis by long terminal repeat (LTR) has been a limitation of retroviral gene transfer [35] since oncogenesis occurred at an unexpected high frequency in the X-SCID gene therapy trial. However, so far, all available data suggest that lentiviral vectors are safe vehicles for ex vivo gene therapy and no adverse events have been reported upon transplantation of lentivirus vector-transduced cells [47]. The major reason for the low genotoxicity of lentiviral vectors may be the lack of transcriptionally active LTR. On the other hand, telomere length maintenance plays critical roles in preventing chromosomal instability and subsequent carcinogenesis. Markedly elevated risks of tumors (about 11 times that of the general population) are observed in patients with dyskeratosis congenita, a disease with very short telomeres caused by germline mutations in the components of the telomerase complex [18]. Mouse models also support the notion that abnormally short telomere length increases the risk of cancers [48]. Recent prospective epidemiological studies have demonstrated that a short telomere is significantly associated with increased cancer incidence and death [49, 50]. Therefore, it is reasonable that lentiviral vector-mediated TERT expression had low tumorigenicity in SHED at late passage, as indicated by no abnormal karyotype, no colony formation in soft agar, and no tumor formation in nude mice. However, our findings need to be extended in SHED over hundreds of passages, and emphasize the caution in the use of TERT-immortalized cells in studies of normal cell biology and in tissue engineering.

There are several limitations in our study. Like most laboratory studies [5], our SHED were maintained and expanded in bovine serum-containing medium, which raises the concern about its clinical application due to the high lot-to-lot variability, risk of contamination, and immune response against xenogenic proteins in bovine serum [51]. Because of the numerous constituents of bovine serum, the development of chemically defined serum-free media with an optimal composition of the few essential factors is only beginning. Thus, bovine serum remains the gold standard medium supplement for laboratory-scale MSC culture and has been used in clinical trials approved by the US Food and Drug Administration [52]. Recent studies have demonstrated that human blood-derived components may be an ideal substitute for bovine serum in the therapeutic application of stem cells. Therefore, SHED expanded in xeno-free media are needed for clinical therapy.

## Conclusions

In this study, we show that a lentiviral TERT gene transduction could establish a stable SHED cell line that is completely multipotential; even after long-term in vitro passaging, no evidence of genetic instability or malignant biological behavior of these cells was observed. These findings provide novel strategies to prevent the senescence and maintain the stemness of ex vivo-maintained SHED for potential clinical therapies, although attention should be paid to the biological behavior of these cells.

## Abbreviations

BaP: benzopyrene
β-GAL: β-galatosidase
Ct: threshold cycle
DSC: dental stem cell
ELISA: enzyme-linked immunosorbent assay
hMSC: human mesenchymal stem cell
LTR: long terminal repeat
MOI: multiplicity of infection
MSC: mesenchymal stem cell
P: passage
PBS: phosphate-buffered saline
PCR: polymerase chain reaction
RT: reverse transcription
SHED: stem cells from human exfoliated deciduous teeth
TERT: telomerase reverse transcriptase
